# The Effects of Probiotics/Synbiotics on Glucose and Lipid Metabolism in Women with Gestational Diabetes Mellitus: A Meta-Analysis of Randomized Controlled Trials

**DOI:** 10.3390/nu15061375

**Published:** 2023-03-12

**Authors:** Jinhao Mu, Xian Guo, Yanbing Zhou, Guoxia Cao

**Affiliations:** 1 Sport Science School, Beijing Sport University, Beijing 100084, China; 2 Key Laboratory of Exercise and Physical Fitness, Ministry of Education, Beijing 100084, China; 3 School of Art, Beijing Sport University, Beijing 100084, China

**Keywords:** gestational diabetes mellitus, GDM, probiotics, pregnancy, gut microbiota

## Abstract

Background: Gestational diabetes mellitus (GDM) is prevalent with lasting health implications for the mother and offspring. Medical therapy is the foundation of GDM management, for achieving optimal glycemic control often requires treatment with insulin or metformin. Gut dysbiosis is a feature of GDM pregnancies, therefore, dietary manipulation of the gut microbiota may offer a new avenue for management. Probiotics are a relatively new intervention, which can reduce the mother’s blood sugar levels and, furthermore, adjust glucose and lipid metabolism in both mother and offspring. Objective: The aim of this systematic review and meta-analysis is to explore the effect of probiotics/synbiotics on glucose and lipid metabolism in women with GDM. Methods: A systematic search of the literature was conducted using the electronic databases Cochrane Library, Web of Science, PubMed, and EBOSCO, published between 1 January 2012 and 1 November 2022. A total of 11 randomized controlled clinical trials (RCTs) were analyzed. The indicators included fasting plasma glucose (FPG), fasting serum insulin (FSI), the homoeostatic model assessment for insulin resistance (HOMA-IR), quantitative insulin sensitivity check index (QUICKI), total cholesterol (TC), HDL cholesterol, LDL cholesterol and triglycerides (TG), the mean weight at end of trial, and gestational weight gain (GWG). Results: Compared with the placebo, probiotics/synbiotics were associated with a statistically significant improvement in FPG (MD = −2.33, 95% CI = −4.27, −0.40, *p* = 0.02), FSI (MD = −2.47 95% CI = −3.82, −1.12, *p* = 0.0003), HOMA-IR (MD = −0.40, 95% CI = −0.74, −0.06, *p* = 0.02), and TC (MD = −6.59, 95% CI = −12.23,−−0.95, *p* = 0.02), while other factors had no significant difference. The subgroup analysis revealed that the kind of supplement led to heterogeneity for FPG and FSI, while heterogeneity was not found for others. Conclusion: Probiotics/synbiotics could control glucose and lipid metabolism in pregnant women with GDM. There was a significant improvement in FPG, FSI, HOMA-IR, and TC. The use of specific probiotic supplementation may be a promising prevention and therapeutic strategy for GDM. However, due to the heterogeneity among existing studies, further studies are warranted to address the limitations of existing evidence and better inform the management of GDM.

## 1. Introduction

Gestational diabetes mellitus (GDM) is defined as any degree of glucose intolerance that is first detected during pregnancy. GDM affects about 12.8% of pregnancies globally [1]. Women with GDM are associated with a high risk of pregnancy outcomes such as eclampsia, shoulder dystocia, cesarean section, while the risk of type 2 diabetes (T2DM), pancreatic cancer, and cardiovascular disease after pregnancy is increased [2,3]. The fetus needs high energy to ensure metabolism and growth in the uterus, of which 80% is provided by glucose [4]. During pregnancies, the mother’s blood glucose could be transported to the fetus through blood vessels in the placenta [5]. The level of fetal glucose could induce secretion of insulin by the fetal pancreas as well as insulin-like growth factors, which play an important role in organ growth and development and fat and glycogen reserves for preventing neonatal glycemic imbalances after birth [4]. In GDM mothers, the persistent high level of blood sugar could result in sustained increase in fetal insulin, which could change hypothalamic response to glucose and lead to long-term mediobasal hypothalamus gliosis as well as insulin resistance [6,7]. This suggests that, later in the newborn’s life, the incidence of congenital malformations, neonatal hypoglycemia, respiratory disorders, cardiometabolic disease, obesity, T2DM, and autism could increase and even neonatal death could occur [8,9,10]. GDM seriously endangers the quality of life for both mothers and children.

It has been proved that an imbalance in gut microbiota is associated with the occurrence of GDM [11]. GDM women had a reduction in alpha diversity when compared to that of normal women at both mid- and late gestation. Meanwhile, in GDM, the Firmicutes/Bacteroidetes (F/B) ratio increases in late pregnancy [12]. These changes in gut microbial composition correlate with fat mass accumulation, increasing blood glucose levels, and insulin resistance [13]. GDM women with gut microbial composition changes are also associated with long-term health burden on their offspring, such as the reduction in alpha diversity and Lactobacillus, and increased Escherichia and Parabacteroides, which could cause an imbalance in the gut flora [14].

In order to prevent the negative influence and decrease the risk factors of GDM, some researchers overviewed the effects of various interventions [15,16,17] (diet, exercise, diet and exercise combined, dietary supplements, pharmaceutical management such as metformin, and the management of other health issues). However, the evidence was of low to moderate quality. Recent research has tried to explore novel, effective, and safe treatment strategies at the population level. Probiotics are defined as “live microorganisms which when administered in adequate amounts confer a health benefit on the host” by the World Health Organization (WHO) [18]. Synbiotics are regarded as indigestible food, which could stimulate and activate bacteria in the digestive tract [19]. It has shown that probiotic/synbiotic supplements are beneficial for pregnancy, which could alleviate insulin resistance and improve lipid metabolism [20]. However, the effect of probiotic/synbiotic supplementation on GDM is controversial, as some randomized controlled trials (RCTs) reported significant improvement in blood glucose control while there was no difference after intervention in other studies [21,22]. Therefore, this meta-analysis was to investigate the interaction between probiotic/synbiotic treatment in relation to glucose and lipid metabolism in GDM.

## 2. Method

### 2.1. Study Protocol

The protocol of this meta-analysis was registered in the International Prospective Register of Systematic Reviews (PROSPERO), ID: CRD42023387754.

### 2.2. Search Strategy

This review followed the 2020 update of the PRISMA statement [23]. A systematic literature search was conducted using the electronic databases Web of Science, PubMed, EBOSCO, and Cochrane Library published between 1 January 2012 and 1 November 2022. The following search terms were used: (pregnan* OR gestation* OR matern* OR obstetric* OR gestational diabetes mellitus OR gestational diabetes OR GDM) AND (probiotic* OR synbiotic* OR lactobacill* OR streptococc* OR bifidobacter* OR saccharomy* OR yeast OR bacteria* OR acidophilus OR ferment* OR microorganism*) AND (glucose* OR insulin). 

### 2.3. Inclusion and Exclusion Criteria

The inclusion criteria were (1) participants were pregnant women with GDM and had no other metabolic diseases, (2) probiotics or synbiotics were used as treatment, (3) participants avoided probiotic- or synbiotic-containing foods and other supplements during intervention, (4) no regular exercise intervention before and during pregnancy, (5) the indicators in studies, related to glucose and lipid metabolism, included, but were not limited to, fasting plasma glucose, fasting serum insulin, total cholesterol, triglycerides, (6) randomized controlled trials and published in English, (7) data reported by means and standard deviation and with the baseline. The exclusion criteria were (1) systematic reviews, case reports, commentaries, meta-analyses and abstracts, (2) animal experiments, (3) probiotics/synbiotics containing unknown bacteria, (4) the intervention group combined other treatment strategies except taking probiotics/synbiotics, (5) data extraction was insufficient.

### 2.4. Data Extraction and Quality Assessment

For each eligible study, the following information was extracted: first author name, published year, country of study, sample size, GDM diagnostic method, the age of participants, intervention duration and frequency, the variety of probiotic/synbiotic bacteria, the dose of supplements, weight at end of trial, gestational weight gain (GWG), fasting plasma glucose (FPG), fasting serum insulin (FSI), the homoeostatic model assessment for insulin resistance (HOMA-IR), quantitative insulin sensitivity check index (QUICKI), total cholesterol (TC), HDL cholesterol, LDL cholesterol, and triglycerides (TG). Two investigators assessed the quality of the included studies by Cochrane Collaboration Risk of Bias. Each study was evaluated from the following perspective: random sequence generation, allocation concealment, blinding, incomplete outcome data, selective outcome reporting, and other bias. The judgement of this perspective was marked as “low risk”, “high risk”, and “unclear risk” of bias.

### 2.5. Statistical Analysis

RevMan v.5.4.1 was used to conduct the meta-analysis. The mean difference and standard deviation were used to report the effect size of continuous data, and a 95% confidence interval (CI) was given, when *p* < 0.05 was considered as a statistically significant difference. The heterogeneity between studies was assessed by I2 and Cochran’s Q test, and *p* value ≤ 0.1 or an I2 ≥ 50% was regarded as showing heterogeneity. When heterogeneity existed, a random-effects model was applied, otherwise a fixed-effects model was chosen. Subgroup analysis was performed to identify the potential causes of heterogeneity. 

## 3. Results

### 3.1. Study Selection

**Figure 1:**
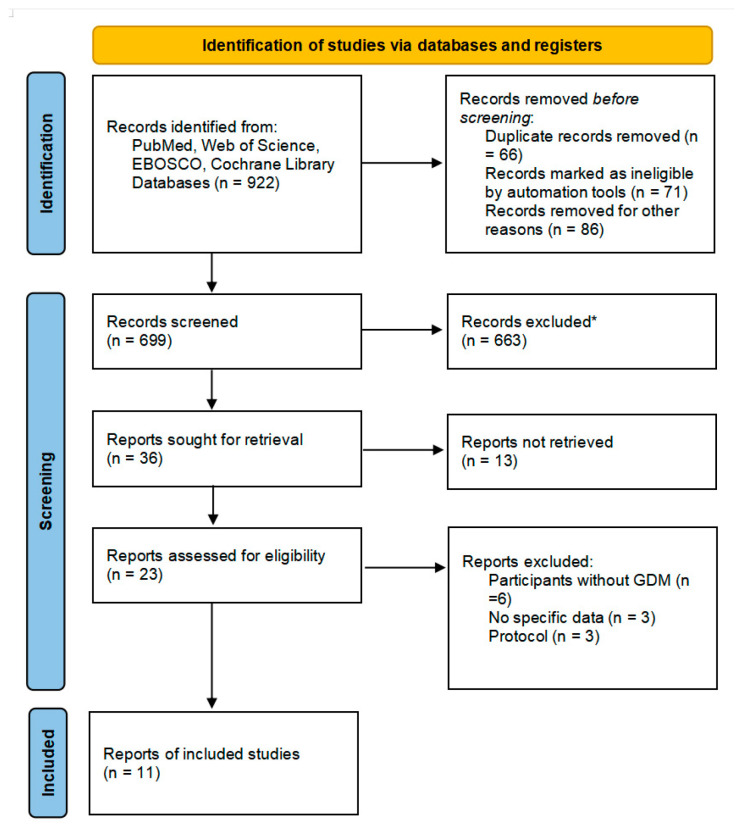
Flow diagram describing inclusion and exclusion criteria. * Automation tools were used; 201 were excluded by automation tools applying filters (animals, review, meta-analysis, and year of publication); and 462 records were excluded by a human.

A total of 922 articles were retrieved by searching the four databases, among which 66 were excluded as duplications and 157 were irrelevant. After screening, 23 were retrieved and assessed for eligibility as part of the selection process. After selection, six studies were excluded as participants did not have GDM, three studies did not have specific data, and three protocols were excluded. Finally, 11 randomized controlled trials met the inclusion criteria and were included in this meta-analysis, as shown in Figure 1.

### 3.2. Description of Included Studies

The articles on RCTs were published from 2012 to 2022. Characteristics of included studies are shown in Table 1. Eight trials were conducted by Iranian researchers, while three were from Thailand, Turkey, and Ireland, respectively. These trials involved 390 participants in probiotics/synbiotics groups and 389 in placebo groups. The mean age of those participants ranged from 26.4 years to 33.5 years. Nine articles diagnosed patients with a “2 h 75 g oral glucose tolerance test”, one article did not mention the diagnostic criteria. Meanwhile, seven of them were based on the American Diabetes Association guidelines [24] and one was based on International Association of Diabetes and Pregnancy Study Groups [25]. The specific criteria are consistent among the different guidelines. One study used a “3 h 100 g oral glucose tolerance test” based on O’Sullivan’s diagnostic criteria [26], and the remaining article did not mention the details of either the diagnostic method or diagnostic criteria. Recent research demonstrated that no convincing evidence showed an advantage, except medical workload and costs [4]. Therefore, this review includes the entire studies. No patients received medicine therapy. The duration of intervention ranged from 4 weeks to 8 weeks. Eight RCTs chose probiotics, including *Lactobacillus acidophilus*, *L. fermentum*, *L.casei*, *L. reuteri*, *L. salivarius*, *L. delbrueckii bulgaricus*, *Bifidobacterium bifidum*, and *Streptococcus thermophilus*, as the intervention method. The remaining three RCTs used synbiotics, including *L. acidophilus*, *L. casei*, *L. fermentum*, *L. gasseria*, *L. plantarum*, *Bififidobacterium bififidum*, *B.longum*, *B. infantis*. Most of them took one capsule a day, only one study adopted two capsules a day. The daily consumption of probiotics/synbiotics varied from 1 × 10^9^ CFU/capsule to 112.5 × 10^9^ CFU/capsule. In our meta-analysis, a variety of after-intervention outcomes were reported including FPG, FSI, HOMA-IR, QUICKI, TC, HDL cholesterol, LDL cholesterol, TG, weight at end of trial, and GWG. The main finding of this meta- analysis is shown in Table 2.

### 3.3. Risk of Bias of Included Studies and Quality of Evidence

**Figure 2:**
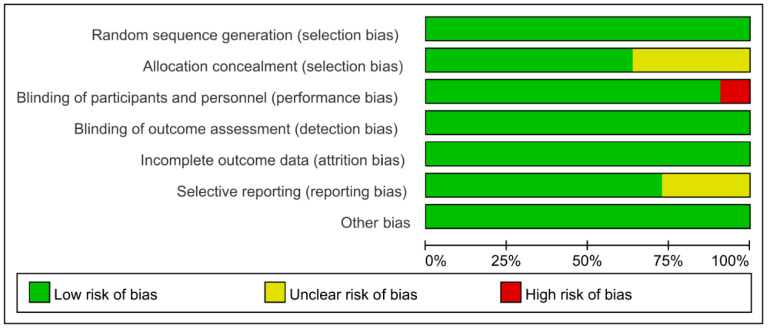
Risk of bias for included studies.

**Figure 3:**
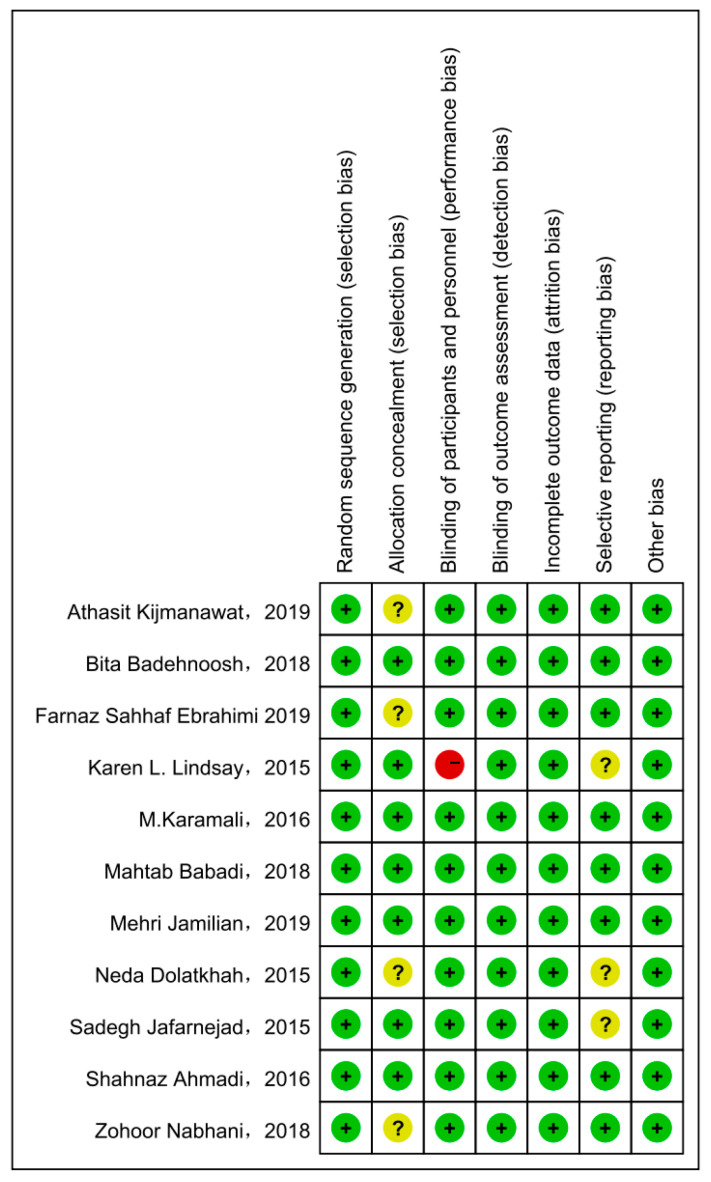
The concrete bias of each study [21,22,27,28,29,30,31,32,33,34,35].

Cochrane Collaboration Risk of Bias was used with the included studies, and objectively evaluated the quality of evidence (i.e., “low risk”, “high risk”, or “unclear risk”). The rating of bias domains for included studies is shown in Figure 2. All studies had low risk in random sequence generation; among them, nine studies used a computer program, while two were conducted by trained staff. Blinding of outcome assessment and incomplete outcome data also showed 100% low risk. Around 50% had low risk in allocation concealment. Only one study had high risk in blinding of participants and personnel. The concrete bias of each study is shown in Figure 3.

### 3.4. Glucose Control

#### 3.4.1. Fasting Plasma Glucose

**Figure 4:**
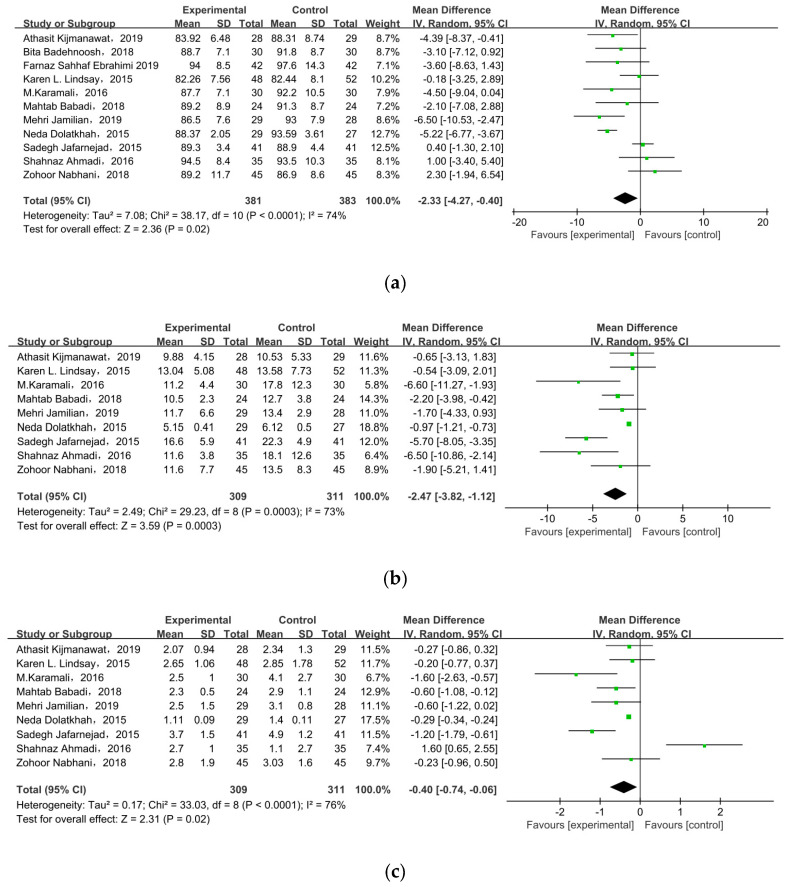

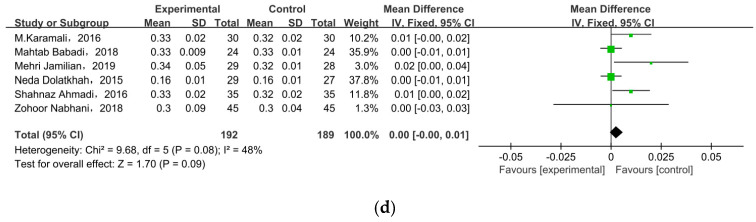
Effect of probiotic supplementation on glucose control in pregnant women with GDM: (**a**) FPG (mg/dL), (**b**) FSI (mU/L), (**c**) HOMA-IR, and (**d**) QUICKI. FPG: fasting plasma glucose; FSI: fasting serum insulin; HOMA-IR: the homoeostatic model assessment for insulin resistance; QUICKI: quantitative insulin sensitivity check index [21,22,27,28,29,30,31,32,33,34,35].

All of the included studies investigated the effect of probiotic/synbiotic supplements on FPG in GDM, as shown in Figure 4a. In the meta-analysis, the mean difference in FPG level was MD = −2.33, 95% CI = −4.27, −0.40, *p* = 0.02, meaning supplements statistically significantly improved FPG level among pregnant women with GDM.

#### 3.4.2. Insulin

Nine of the eleven studies reported FSI as an outcome. The overall pooled estimate of the mean difference in FSI was MD = −2.47, 95% CI = −3.82, −1.12, *p* = 0.0003 (Figure 4b), indicating the intervention could improve FSI in women with GDM. HOMA-IR was observed in nine studies. After meta-analysis, we found a significant effect favoring probiotics/synbiotics, as shown in Figure 4c (MD = −0.40, 95% CI = −0.74, −0.06, *p* = 0.02). Six studies measured QUICKI, but there was no significant difference between the two groups (MD = 0.00, 95% CI = 0.00, 0.01, *p* = 0.09) (Figure 4d).

### 3.5. Lipid Profiles

**Figure 5:**
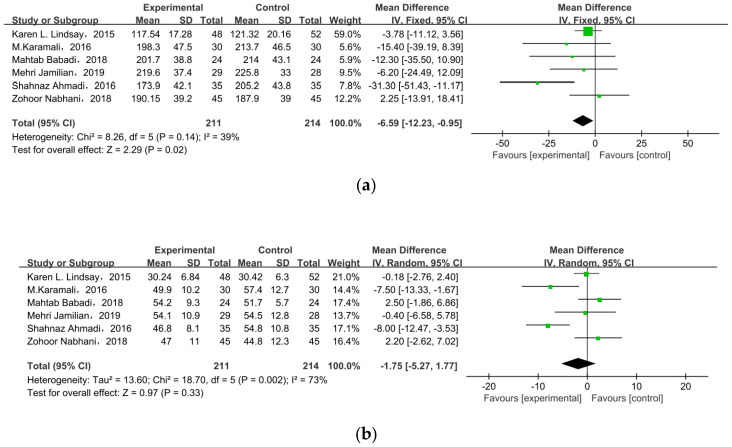

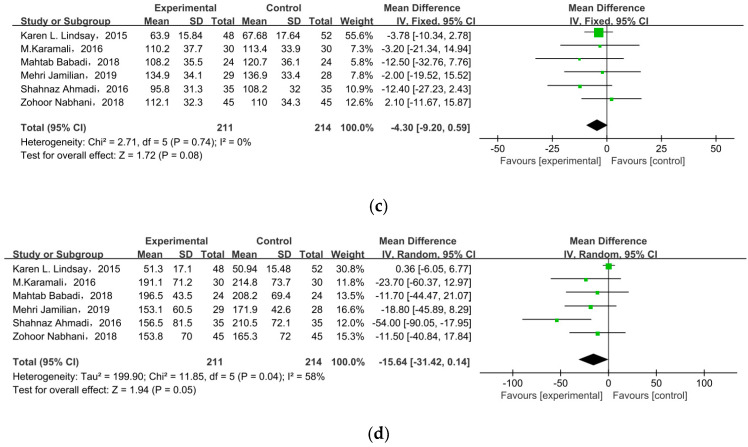
Effect of probiotic supplementation on lipid profiles in pregnant women with GDM: (**a**) TC (mg/dL), (**b**) HDL cholesterol (mg/dL), (**c**) LDL cholesterol (mg/dL), and (**d**) TG (mg/dL). TC: total cholesterol; TG: triglycerides [22,29,30,31,34,35].

Six studies researched lipid profiles, including TC, HDL, LDL, and TG (Figure 5). All data were pooled for meta-analysis, and only TC was significantly reduced in pregnant women with GDM after receiving supplement therapy (MD = −6.59, 95% CI = −12.23, −0.95, *p* = 0.02) (Figure 5a). On the contrary, the other observed indexes described no obvious change between treatment groups and control groups: HDL cholesterol (MD = −1.75, 95% CI = −5.27, 1.77, *p* = 0.33) (Figure 5b), LDL cholesterol (MD = −4.30, 95% CI = −9.20, 0.59, *p* = 0.08), TG (MD = −15.64, 95% CI = −31.42, 0.14, *p* = 0.05) (Figure 5c).

### 3.6. Body Weight

#### 3.6.1. Weight at End of Trial

**Figure 6:**
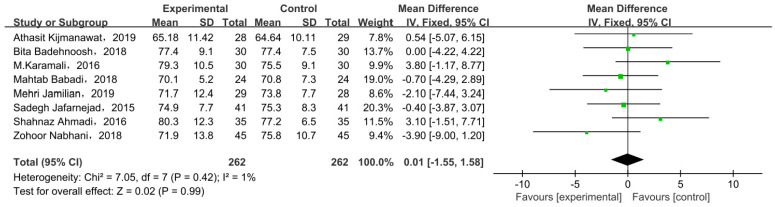
Effect of probiotic supplementation on weight at end of trial in pregnant women with GDM [21,27,29,30,31,33,34,35].

Eight studies compared body weight at end of trial with two types of intervention. There was no significant difference in body weight between the probiotic/synbiotic and placebo groups at the end of test in all included studies, as shown by the meta-analysis (Figure 6). The mean difference in body weight was 0.01 (95% CI = −1.55, 1.58, *p* = 0.99).

#### 3.6.2. Gestational Weight Gain

**Figure 7:**
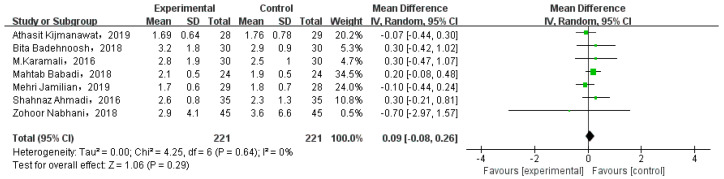
Effect of probiotic supplementation on gestational weight gain in pregnant women with GDM [21,27,29,30,31,34,35].

Eight studies involved GWG. While one of them compared the weight gain at different periods, and the data could not be extracted. Thus, we analyzed seven studies, as shown in Figure 7. There was no significant gestational weight gain between two types of intervention (MD = 0.09, 95% CI = −0.08, 0.26, *p* = 0.29).

### 3.7. Subgroup Analysis

Substantial heterogeneity was observed in the meta-analyses for FPG (I^2^ = 74%, *p* < 0.0001), FSI (I^2^ = 73%, *p* = 0.0003), HOMA-IR (I^2^ = 76%, *p* ≤ 0.0001), HDL cholesterol (I^2^ = 73%, *p* = 0.002), and TG (I^2^ = 58%, *p* = 0.04). We grouped duration and kind of supplement to investigate the potential reason for heterogeneity. The *p*-value and heterogeneity for different groups are shown in Table 3. The results showed that the kind of supplement led to heterogeneity for FPG and FSI, while we did not find the main cause of heterogeneity for others.

## 4. Discussion

This review included 11 RCTs and 779 participants to assess the effect of probiotic/synbiotic supplements on improving glucose and lipid metabolism in women with GDM. We assessed the level of FPG, FSI, HOMA-IR, and QUICKI to explain the effect of supplements on glucose control. TC, HDL cholesterol, LDL cholesterol, and TG were used to investigate the effect of intervention on lipid metabolism. The weight at end of trial and gestational weight gain were used to reflect the maternal fat accumulation during pregnancy. This meta-analysis found the intervention improved glucose and lipid metabolism in terms of FPG (MD = −2.33, *p* = 0.02), FSI (MD = −2.47, *p* = 0.0003), HOMA-IR (MD = −0.40, *p* = 0.02), and TC (MD = −6.59, *p* = 0.02) in GDM women.

GDM is usually caused by β-cell impairment and insulin resistance. During the second and third trimesters of pregnancy, anti-insulin hormones such as estrogen, progesterone, and cortisol promote the development of insulin resistance [37]. In order to maintain the normal metabolism of glucose, the amount of insulin needs to be increased accordingly. During gestational diabetes, β-cells fail to secrete insulin for the demands of pregnancy, and, when combined with reduced insulin sensitivity, this results in hyperglycemia [38]. GDM is also related to reduced adipocyte differentiation and increased adipocyte size. Furthermore, this change is combined with insulin resistance, which leads to lipid deposition in the liver and skeletal muscle, thus aggravating the condition of GDM [38,39].

In GDM patients, a high level of body fat and intake of high-fat nutrition could change the normal gut microbial composition. This could increase Firmicutes and Faecalibacterium that produce butyrate, and result in overexpression of short chain fatty acids (SCFAs) [20]. Excess SCFAs increase the lipid storage in skeletal muscle and liver, thus glucose and lipid metabolism disorders develop [40]. Probiotics could perform a straightforward and important role in the intestinal mucosal barrier, which increases glucose tolerance, while reversing the gut flora imbalance induced by GDM [41]. Probiotics also regulate the level of SCFAs. When the SCFAs are within the normal range, they could regulate glucose metabolism, lowering the pH of the lumen while preventing pathogen growth. The intestinal epithelial cells could be activated by SCFAs through the cells’ G protein-coupled receptor 41 (GPR41) and GPR43, and the colonic epithelial intestinal expression of peptide YY and glucagon-like peptide-1 (GLP-1) hormones will thereby be increased [42,43].

Thus, transit time of food in the intestine could slow and insulin sensitivity could increase. By tight junction protein transcription regulation and glucagon-like peptide-2 (GLP-2) production enhancement, SCFAs decrease intestinal permeability and may reduce inflammation in colonic epithelial cells [44]. Moreover, SCFAs could be recognized by free fatty acid receptor-2 (FFA-2), and research has shown the synergistic effect between SCFAs, FFA-2, and gut microbiota in gestational glucose homeostasis [45]. Inulin is a common synbiotic. Taking inulin could improve glucose metabolism dysregulation and overweight, decrease the Firmicutes/Bacteroidetes ratio (F/B), as well as increase Bifidobacterium abundance [46,47]. Consistent with Shahnaz Ahmadi et al.’s [34]. Findings, we found that after 6 weeks of synbiotic supplements the FSI and HOMA-IR were significantly reduced. In this meta-analysis, six RCTs reported lipid profiles, and after intervention TC showed a significantly decrease. However, some other clinical studies have produced conflicting results, with taking probiotics or placebo in the first trimester of pregnancy having no effect on the lipid levels in the third trimester [22]. Beneficial gut bacteria are increased after taking probiotics/synbiotics. Thus, with the production of secondary bile acids that are not available for enterohepatic recirculation, those gut flora have a favorable impact on lipid metabolism. Next, the liver must produce new bile acids from circulating cholesterol [48]. The population with abnormal gut microbes and glucose and lipid metabolism may have insulin resistance, increasing the onset risk of GDM and the mother’s body weight. Continuous hyperglycemia in pregnant women can affect the fetal insulin levels, thereby accelerating fetal weight gain. Probiotics/synbiotics could lower maternal insulin resistance and restore gut flora to keep the mother’s weight in the normal range, as well as reduce the risk of eclampsia, shoulder dystocia, T2DM, and other GDM-related diseases [49]. The offspring absorbs nutrients through the placenta, further reducing the incidence of neonatal hypoglycemia, obesity, T2DM, and autism [50]. However, our included RCTs showed no difference between treatment and placebo on HDL cholesterol and LDL cholesterol. We hypothesized that a duration of intervention longer than 8 weeks may have generated outcomes with larger effect sizes.

In the current study, we also found substantial heterogeneity among FPG, FSI, HOMA-IR, HDL cholesterol, and TG. The subgroup analyses on FPG indicate that probiotic supplements may lead to great improvement in the GDM population. There was no significant difference in FSI with more than 8 weeks’ nutritional intervention, as only two relevant RCTs were included and the data were insufficient. In the subgroup analyses of intervention methods, synbiotics failed to reduce the HOMA-IR. For HDL and TC, the subgroup analysis revealed no significant differences. Prespecified subgroup analyses according to duration, intervention type, and dose showed that the heterogeneity was substantially reduced or even disappeared in a few subgroups, but persisted in most others. A possible reason is that the potential effect modification by the two subgroup factors could not be effectively investigated by our subgroup analyses. For example, the dose of the treatments varied from 1 × 10^9^ CFU/capsule to 112.5 × 10^9^ CFU/capsule, and some only had the range of the dose rather than the specific number. Another possible explanation is that the substantial heterogeneity may relate to the interaction of duration, intervention type, and dose. Additionally, this may be caused by other factors, e.g., the mean age and test method. In addition, most of the included studies are from Iran. Subsequent studies could increase duration (>8 weeks), include other racial backgrounds, and confirm the quantity of the dose, to reduce the interference factors.

## 5. Strength and Limitation

Our meta-analysis has several strengths. First, this study consists of multiple articles, to include more data for analysis and increase the reliability of the results. Second, most of the included RCTs were high quality with random sequence generation and double-blinding methods, so the data are reliable. Third, studies whose population was limited to women with GDM made the results more representative. However, the current study has a few limitations. First, the numbers of participants in the included RCTs were relatively small, ranging from 20 to 60. Second, eight RCTs were conducted in Iran, which means the research may show publication bias and the conclusions may not be applicable to populations from other racial backgrounds and countries. Third, we did not assess the effect of probiotic/synbiotic supplementation on infants and failed to capture the long-term effect for mothers and their offspring. Furthermore, the types and durations of probiotics/synbiotics were not consistent in the different studies that were included in this analysis. To identify the effect of intervention duration and type, more RCTs must be conducted. Overall, the glucose and lipid metabolism-related indicators were significantly improved, which can indicate that probiotic/synbiotic supplements may be considered as an adjunct treatment for GDM patients.

## 6. Conclusions

Probiotic/synbiotic could improve glucose and lipid metabolism in pregnant women with GDM. The use of specific probiotic supplementations containing Lactobacillus acidophilus and Bifidobacterium bifidum (>1 × 10^6^ CFU/g) may be a promising prevention and therapeutic strategy for GDM, as they could directly act on the intestinal mucosal barrier and restore the gut flora balance. However, due to the heterogeneity among existing studies, further studies are warranted to address the limitations of existing evidence and better inform the management of GDM.

## Figures and Tables

**Table 1:** Characteristics of included studies.

Number	Author, Year	Country	Diagnostic Method	Diagnostic Criteria	Duration (Weeks)	Frequency of Treatment	Sample Size (Probiotic/Placebo)	Mean Age (Years) (Probiotic/Placebo)	Intervention	Outcomes
1	Athasit Kijmanawat 2019 [21]	Thailand	2 h 75 g oral glucose tolerance test (OGTT)	Based on International Association of Diabetes and Pregnancy Study Groups	4	1 capsule/day	28/29	32.50 ± 5.02/30.72 ± 5.05	1 × 10^9^ CFU *Lactobacillus acidophilus*, *Bifidobacterium bifidum*	①, ②, ③, ⑨, ⑩
2	Bita Badehnoosh 2018 [27]	Iran	2 h 75 g oral glucose tolerance test (OGTT)	Based on the American Diabetes Association guidelines	6	1 capsule/day	30/30	28.8 ± 5.4/27.8 ± 3.7	2 × 10^9^ CFU/g each; *Lactobacillus acidophilus*, *Lactobacillus casei*, *Bifidobacterium bifidum*	①, ⑨, ⑩
3	Farnaz Sahhaf Ebrahimi 2019 [28]	Iran	2 h 75 g oral glucose tolerance test (OGTT)	Unknown	8	300 mg/day	42/42	31.64 ± 5.97/31.61 ± 5.49	1 × 10^6^ CFU/g; *Lactobacillus acidophilus*, *Bifdobacterium lactis*	①
4	Karen L. Lindsay 2015 [22]	Ireland	3 h 100 g oral glucose tolerance test	Based on O’Sullivan’s diagnostic criteria	4–6	1 capsule/day	57/58	33.5 ± 5.0/32.6 ± 4.5	1 × 10^9^ CFU/g; *Lactobacillus salivarius* UCC118	①, ②, ③, ⑤, ⑥, ⑦, ⑧
5	Karamali 2016 [29]	Iran	2 h 75 g oral glucose tolerance test (OGTT)	Based on American Diabetes Association guidelines	6	1 capsule/day	30/30	31.8 ± 6.0/29.7 ± 4.0	2 × 109 CFU/g; *L. acidophilus*, *L. casei*, *B. bifidum*	①, ②, ③, ④, ⑤, ⑥, ⑦, ⑧, ⑨, ⑩
6	Mahtab Babadi 2018 [30]	Iran	2 h 75 g oral glucose tolerance test (OGTT)	Based on the American Diabetes Association guidelines	6	1 capsule/day	24/24	28.8 ± 4.3/29.0 ± 4.2	2 × 10^9^ CFU/g; *Lactobacillus acidophilus*, *Lactobacillus casei*, *Bifidobacterium bifidum*, *Lactobacillus fermentum*	①, ②, ③, ④, ⑤, ⑥, ⑦, ⑧, ⑨, ⑩
7	Mehri Jamilian 2019 [31]	Iran	2 h 75 g oral glucose tolerance test (OGTT)	Based on the American Diabetes Association guidelines	6	1 capsule/day	29/28	31.2 ± 5.9/29.9 ± 3.7	2 × 10^9^ CFU/g each; *Lactobacillus acidophilus*, *Bifidobacterium bifidum*, *L. reuteri*, *Lactobacillus fermentum*	①, ②, ③, ④, ⑤, ⑥, ⑦, ⑧, ⑨, ⑩
8	Neda Dolatkhah 2015 [32]	Turkey	By either a gynecologist or an internal medicine specialist	Unknown	8	1 capsule/day	29/27	28.14 ± 6.24/26.48 ± 5.23	>1 × 10^9^ CFU/g each; *Lactobacillus acidophilus* LA-5, *Bifidobacterium* BB-12, *Streptococcus thermophilus* STY-31, *Lactobacillus delbrueckii bulgaricus* LBY-27	①, ②, ③, ④,⑩
9	Sadegh Jafarnejad 2016 [33]	Iran	2 h 75 g oral glucose tolerance test (OGTT)	Based on the American Diabetes Association guidelines	8	2 capsule/day	41/41	32.4 ± 3.1/31.9 ± 4.0	112.5 × 10^9^ CFU/capsule *Streptococcus thermophilus*, *Bifidobacterium breve*, *Bifidobacterium longum*, *Bifidobacterium infantis*, *Lactobacillus acidophilus*, *Lactobacillus plantarum*, *Lactobacillus paracasei*, and *Lactobacillus delbrueckii* subsp. *Bulgaricus* and microcrystalline cellulose, stearic acid, magnesium stearate, vegetable material (hydroxypropyl methylcellulose), silicon dioxide	①, ②, ③, ⑨
10	Shahnaz Ahmadi 2016 [34]	Iran	2 h 75 g oral glucose tolerance test (OGTT)	Based on the American Diabetes Association guidelines	6	1 capsule/day	35/35	28.5 ± 5.8/28.7 ± 3.4	2 × 10^9^ CFU/g each *Lactobacillus acidophilus*, *Lactobacillus casei*, *Bifidobacterium bifidum* plus 0.8 g inulin	①, ②, ③, ④, ⑤, ⑥, ⑦, ⑧, ⑨, ⑩
11	Zohoor Nabhani 2018 [35]	Iran	2 h 75 g oral glucose tolerance test (OGTT)	Based on the American Diabetes Association guidelines	6	500 mg/day	45/45	29.4 ± 5.8/30.3 ± 5.6	*L. acidophilus* (5 × 10^10^ CFU/g), *L. plantarum* (1.5 × 10^10^ CFU/g), *L. fermentum* (7 × 10^9^ CFU/g), *L. gasseri* (2 × 10^10^ CFU/g), and 38.5 mg of FOS	①, ②, ③, ④, ⑤, ⑥, ⑦, ⑧, ⑨, ⑩

① FPG, ② FSI, ③ HOMA-IR, ④ QUICKI, ⑤ TC, ⑥ HDL cholesterol, ⑦ LDL cholesterol, ⑧ TG, ⑨ weight at end of trial, ⑩ GWG.

**Table 2:** The main findings of this meta-analysis [36].

Outcomes	Significant Improvement in Outcomes	*p*
FPG (mg/dL)	Y	0.02
FSI (mU/L)	Y	0.0003
HOMA-IR	Y	0.02
QUICKI	N	0.09
TC (mg/dL)	Y	0.02
HDL cholesterol (mg/dL)	N	0.33
LDL cholesterol (mg/dL)	N	0.08
TG (mg/dL)	N	0.05
Weight at end of trial (kg)	N	0.99
GWG (kg)	N	0.29

Y: Yes; N: No.

**Table 3:** The results of subgroup analysis according to duration, intervention type, and dose.

Subgroup	Studies	Participants	Mean Difference (95% CI)	Heterogeneity (I^2^%)	*p*
FPG					
Duration < 8weeks	8	557	−2.14 (−4.23, −0.05)	53	0.05
Duration ≥ 8weeks	3	222	−2.73 (−7.08, 1.63)	91	0.22
Probiotic	8	537	−3.77 (−5.37, −2.17)	39	<0.00001
Synbiotic	3	242	0.70 (−0.79, 2.19)	0	0.35
Dose < 2 × 10^9^ CFU/g	4	312	−3.40 (−6.10, −0.70)	68	0.01
Dose ≥ 2 × 10^9^ CFU/g	7	467	−1.63 (−3.97, 0.70)	63	0.17
FSI					
Duration < 8weeks	7	497	−2.20 (−3.61, −0.79)	43	0.002
Duration ≥ 8weeks	2	138	−3.18 (−7.81, 1.44)	94	0.18
Probiotic	6	393	−1.37 (−2.29, −0.45)	36	0.003
Synbiotic	3	242	−4.65 (−7.33, −1.97)	51	0.0007
Dose < 2 × 10^9^ CFU/g	3	228	−0/96(-1.20, −0.72)	0	<0.00001
Dose ≥ 2 × 10^9^ CFU/g	6	407	−3.70 (−5.51, −1.89)	57	<0.0001
HOMA-IR					
Duration < 8weeks	7	497	−0.34 (−0.58, −0.10)	75	0.006
Duration ≥ 8weeks	2	138	−0.30 (−0.35, −0.24)	89	<0.00001
Probiotic	6	393	−0.30 (−0.35, −0.25)	43	<0.00001
Synbiotic	3	242	−0.36 (−0.78, 0.05)	92	0.08
Dose < 2 × 10^9^ CFU/g	3	228	−0.29 (−0.34, −0.24)	0	<0.00001
Dose ≥ 2 × 10^9^ CFU/g	6	407	−0.57 (−0.84, −0.30)	83	<0.0001
HDL cholesterol					
Probiotic	4	280	−0.96 (−4.44, 2.52)	60	0.59
Synbiotic	2	160	−2.94 (−12.94, 7.05)	89	0.56
TG					
Probiotic	4	280	−5.15 (−15.79, 5.49)	19	0.34
Synbiotic	2	160	−31.41 (−72.97, 10.16)	69	0.14

## Data Availability

The protocol was registered in the International Prospective Register of Systematic Reviews (PROSPERO): registration ID CRD42023387754.
